# Muscle loss phenotype in COPD is associated with adverse outcomes in the UK Biobank

**DOI:** 10.1186/s12890-024-02999-7

**Published:** 2024-04-17

**Authors:** Amy H. Attaway, Rocio Lopez, Nicole Welch, Annette Bellar, Umur Hatipoğlu, Joe Zein, Marielle PKJ Engelen, Srinivasan Dasarathy

**Affiliations:** 1 Departments of Pulmonary Medicine, Cleveland Clinic, Cleveland, OH USA; 2 Center for Populations Health Research, Cleveland Clinic, Cleveland, OH USA; 3 Inflammation and Immunity, Cleveland Clinic, Cleveland, OH USA; 4 Gastroenterology and Hepatology, Cleveland Clinic, Cleveland, OH USA; 5 Department of Medicine, Mayo Clinic, Scottsdale, AZ USA; 6 Department of Kinesiology, Texas A&M University, College Station, TX USA; 7 Department of Inflammation and Immunity, Lerner Research Institute, 9500 Euclid Avenue, Cleveland, OH 44195 USA

**Keywords:** Cachexia, Chronic obstructive pulmonary disease, Epidemiology, Sarcopenia, Skeletal muscle loss

## Abstract

**Background:**

Chronic obstructive pulmonary disease (COPD) is a chronic inflammatory disorder with systemic consequences that can cause a muscle loss phenotype (MLP), which is characterized by the loss of muscle mass, muscle strength, or loss of both muscle and fat mass. There are limited data comparing the individual traits of MLP with clinical outcomes in a large unbiased cohort of COPD patients. Our aim was to determine the proportion of patients who met criteria for MLP in an unbiased sample of COPD patients at the population-level. We also determined if specific MLP features were associated with all-cause and COPD-related mortality.

**Methods:**

A retrospective population-based cohort analysis of the UK Biobank was performed. COPD was defined by a FEV1/FVC ratio < 0.7, physician established diagnosis of COPD, or those with a COPD-related hospitalization before baseline assessment. MLP included one or more of the following: 1) Low fat-free mass index (FFMI) on bioelectric impedance analysis (BIA) or 2) Appendicular skeletal muscle index (ASMI) on BIA, 3) Low muscle strength defined by handgrip strength (HGS), or 4) Low muscle and fat mass based on body mass index (BMI). Cox regression was used to determine the association between MLP and all-cause or COPD-related mortality. All models were adjusted for sex, age at assessment, ethnicity, BMI, alcohol use, smoking status, prior cancer diagnosis and FEV1/FVC ratio.

**Results:**

There were 55,782 subjects (56% male) with COPD followed for a median of 70.1 months with a mean(± SD) age at assessment of 59 ± 7.5 years, and FEV1% of 79.2 ± 18.5. Most subjects had mild (50.4%) or moderate (42.8%) COPD. Many patients had evidence of a MLP, which was present in 53.4% of COPD patients (34% by ASMI, 26% by HGS). Of the 5,608 deaths in patients diagnosed with COPD, 907 were COPD-related. After multivariate adjustment, COPD subjects with MLP had a 30% higher hazard-ratio for all-cause death and 70% higher hazard-ratio for COPD-related death.

**Conclusions:**

Evidence of MLP is common in a large population-based cohort of COPD and is associated with higher risk for all-cause and COPD-related mortality.

**Supplementary Information:**

The online version contains supplementary material available at 10.1186/s12890-024-02999-7.

## Introduction

Chronic obstructive pulmonary disease (COPD) is a progressive illness and the third most common cause of death globally [1]. Loss of skeletal muscle mass or contractile strength (i.e., sarcopenia) is a major contributor to morbidity and mortality in COPD [2, 3]. While loss of muscle mass or strength is common in COPD and associated with advanced disease [4, 5], others have reported that sarcopenia may present early or independent of COPD severity [6, 7]. Also, the individual traits (i.e., reductions in muscle mass, strength, or physical performance) may present in patients prior to meeting the complete criteria for sarcopenia [8]. Our previous analyses of administrative data showed that a range of diagnoses related to muscle loss which we have termed a muscle loss phenotype (MLP), can reliably and consistently predict adverse outcomes in COPD patients [2].

Previous studies have shown that reductions in physical performance have been associated with mortality in COPD patients [9]. While COPD exacerbation rate and dyspnea burden predict risk for future exacerbations [10], early pulmonary rehabilitation after a COPD exacerbation is one of the few therapies to improve survival in these patients [11]. These findings suggest that incorporating measures of muscle mass and muscle strength into the evaluation has the potential to improve the clinical outcomes of COPD patients.

The UK Biobank is a large-scale biomedical database with clinical and anthropometric data on over half a million participants. Using a large unbiased cohort like the UK Biobank allows for significant phenotyping of an unbiased cohort of COPD patients, with measures of total muscle mass (i.e. fat free mass index or FFMI), peripheral muscle mass (i.e. appendicular skeletal muscle index or ASMI), muscle strength (i.e. handgrip strength or HGS), or combined muscle and fat mass (i.e. body mass index or BMI). Previous studies have noted that both low FFMI and low BMI predict mortality in COPD patients [12], while others have suggested that low ASMI may be a better predictor of outcomes in COPD patients [13]. While low BMI typically involves loss of muscle mass and fat mass, it is the most frequently used measure used in clinical practice to diagnose muscle loss and has been incorporated into COPD prognostication [14]. Loss of muscle strength occurs more rapidly than muscle mass in an aged population, suggesting that HGS could predict a decline in muscle contractile function prior to loss of muscle mass [15]. Our goal was to determine the proportion of patients who met criteria for a muscle loss phenotype or MLP (as defined by low FFMI, ASMI, HGS, or BMI) in an unbiased sample of COPD patients at the population-level, and determine which MLP features were associated with all-cause and COPD-related mortality in the UK Biobank.

## Materials and methods

### Data description

A retrospective cohort analysis was performed using data from adult participants in the UK Biobank baseline survey (2006–2010). UK Biobank participants have previously been described in detail [16]. The UK Biobank is a cohort of 502,536 participants (age 37-73y) from all across the United Kingdom, who lived within 25 miles of 22 assessments centers in England, Wales, and Scotland. Participants answered questions about their illnesses by completing touchscreen questionnaires or verbal interviews, underwent a physical examination, and provided biologic samples. A written informed consent was obtained from all participants. Spirometry measurements were available on 453,724 participants. Access to the dataset was obtained and analysis performed after obtaining approval from the Institutional Review Board at the Cleveland Clinic (IRB #20–446). Data analysis was performed between Sept 1, 2021 to Dec 1, 2022.

### Definitions

We defined COPD as a FEV1/FVC ratio < 0.7 using previously defined criteria [17]. Spirometric COPD was defined by pre-bronchodilator spirometry as the UK Biobank participants did not undergo post-bronchodilator spirometry testing, which is consistent with other large scale epidemiologic studies like the Copenhagen Heart Study [12]. Subjects who reported a previous diagnosis of COPD by a physician and those with a COPD-related hospitalization prior to the baseline assessment were also included.

#### Measurements

All measurements were obtained as detailed in the UK Biobank protocols [18]. BMI was defined as weight/[height (in meters)]^2^. Weight, fat-free mass and fat mass were determined using bioelectrical impedance analysis (BIA) with a Tanita BC-418 MA body composition analyzer (Tanita Europe, Amsterdam, The Netherlands). Prior to BIA, participants were instructed to remove shoes and heavy clothing. Standing height was measured using a Seca202 height measure. HGS was measured once each for the right and left hands using a Jamar J00105 hydraulic hand dynamometer (Sammons Preston, Bolingbrook, IL) after participants were instructed on its usage by trained personnel. The two measures were averaged together per subject to provide a single handgrip strength. Physical activity was assessed using questions adapted from a validated short International Physical Activity Questionnaire (IPAQ) [19]. Spirometry was measured using the Pneumotrac 6800 spirometer (Vitalograph Ltd. UK, Buckingham, England) with 3 measurements over 6 min to allow for at least 2 technically acceptable measurements. The percentage of predicted FEV1 was calculated as previously described [20]. We defined “evidence of MLP” in patients who satisfied one or more individual measures of skeletal muscle mass or strength as defined in Supp Table 1

In addition, ICD-9-CM and ICD-10-CM codes were used to determine hospitalizations related to COPD, MLP, or select lung conditions and ICD-10-CM codes were used to determine if deaths were related to COPD or other causes (Supp Table 2).

### Statistical analysis

Continuous variables were evaluated for normality using the Shapiro–Wilk test. Normally distributed continuous measures were summarized using means and standard deviations (SDs) and were compared between subjects with and without MLP using one or more of our predefined criteria using t-tests. Non-normally distributed continuous and ordinal measures were summarized using medians, 25th and 75th percentiles and were compared between groups using Kruskal–Wallis tests. Categorical factors were compared using Pearson’s chi-square tests. Standardized differences between the 2 groups are also reported.

We used Cox regression to determine the association between MLP and all-cause death. In addition, we used a competing risks regression model to evaluate the association between MLP and COPD-related death with death due to other causes [21]. Competing risk analysis avoids the bias of informative censoring. Follow-up was defined as the time between the initial UK biobank assessment and date of death. Follow-up time ranged between 42 to 82 months with a median of 71 months [P25, P75: 66.6, 75.0]. COPD subjects were censored at time of loss-to-follow-up or June 30, 2020 (the last time data was accessed). In addition, we used zero-inflated negative binomial regression to assess the association between MLP and number of hospitalizations (all cause). Zero-inflated binomial regression is performed to improve modeling when there are excess zeros in the outcome [22], and assumes: 1) the subject has been hospitalized at least once vs. never been hospitalized or 2) if they have been hospitalized, then the number of hospitalizations is quantified. Covariables known to affect risk for MLP were chosen a priori based on our review of literature [2, 23, 24] and included sex, age at assessment, ethnicity (British White vs. Other), BMI, current alcohol use, smoking status (current vs former vs never), prior cancer diagnosis, and FEV1/FVC ratio. We used FEV1/FVC ratio to adjust for severity of COPD instead of FEV1% predicted because FEV1% predicted is adjusted for age, height, gender and ethnicity [25], and these covariables are already incorporated into our model or our definitions for MLP.

We used multiple imputation with the Markov Chain Monte Carlo method and a single chain to impute 5 datasets with complete data. The multiple imputation included the covariables listed above. All models were fitted on each of the 5 imputed datasets and parameter estimates were combined using SAS MIanalyze. All tests were two-tailed and performed at a significance level of 0.05. Analyses were performed using SAS 9.4 software (SAS Institute, Cary, NC).

## Results

### Patient characteristics

**Fig. 1:**
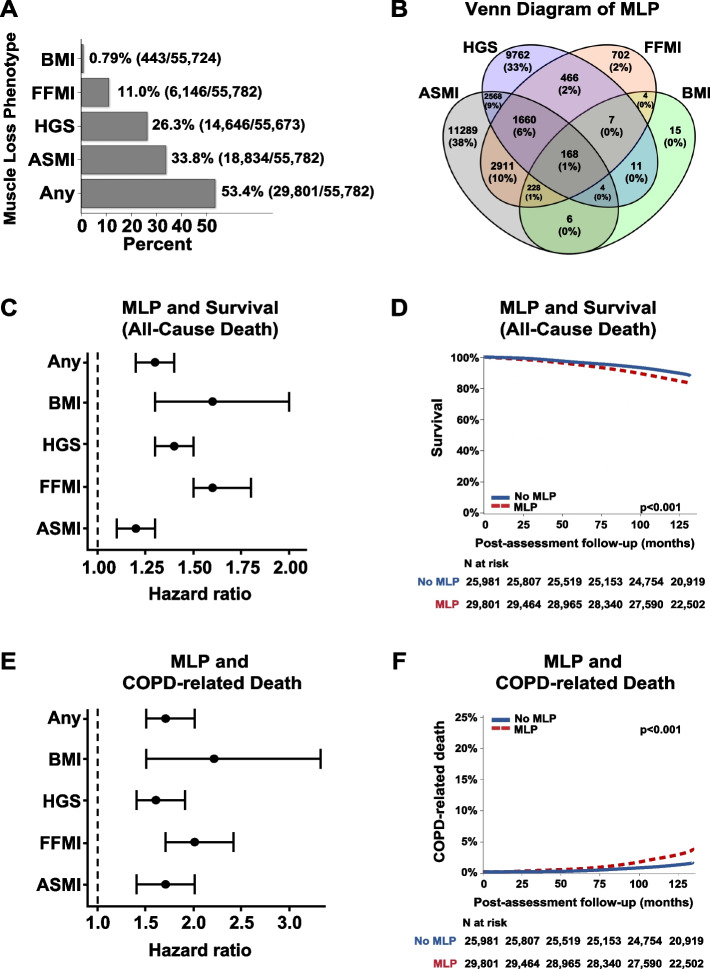
Association of muscle loss phenotype with clinical outcomes. **A** Proportion of patients with evidence of muscle loss phenotype (MLP) based on individual and combined criteria. Overall, 53.4% of subjects with chronic obstructive pulmonary disease (COPD) met at least one criterion for MLP. Proportion of patients with evidence of MLP was lowest using body mass index (BMI) criteria and highest using appendicular skeletal muscle index (ASMI) criteria. **B** Venn diagram of criteria. A Venn diagram using ellipses demonstrates all possible relationships between different definitions of MLP and provides information on the proportion of patients that met one or more criteria for MLP (see Supplementary Table 1 for MLP definitions). The greatest overlap between two definitions was FFMI-defined MLP and ASMI-defined MLP (*n* = 2911, or 10%). The greatest overlap between three definitions was FFMI-defined MLP, ASMI-defined MLP, and HGS-defined MLP (*n* = 1660, or 6%). **C** Hazard ratios from Cox proportional analysis of MLP and all-cause mortality. Fat free mass index (FFMI) and BMI were most associated with all-cause mortality. **D** Cox-regression analysis of MLP and survival (all-cause mortality). Evidence of MLP (defined by at least one criterion) was associated with increased risk for all-cause mortality over time. **E** Hazard ratios from Cox proportional analysis of MLP and COPD-related death. FFMI was most associated with COPD-related mortality. **F** Cox-regression analysis of MLP criteria and COPD-related death. Evidence of MLP was associated with increased COPD-related mortality over time. MLP (defined by at least one criteria) was associated with increased risk for COPD-related death over time

We identified 55,782 (11.1%) subjects with COPD in the cohort (Supp Figure 1). Average age (± SD) at assessment was 59 ± 7.5 years and 56% of subjects were male. The majority of COPD patients were early stage COPD, with an average FEV1% of 79.2 ± 18.5%, and the majority of patients were either mild (50.4%) or moderate (42.8%) based on GOLD stage [17]. Consistent with previous reports from the UK Biobank, the study population was predominantly British White ethnicity (94.1%). A higher proportion of patients with evidence of MLP were current smokers (21% versus 17.2%, *p* < 0.001). In the MLP cohort, pack years of smoking (27.5 [14.8, 42.0] vs. 25.5 [14.0, 39.4], *p* < 0.001; median, 1st and 3rd interquartile range) and diagnosis of cancer (9.3% vs 7.7%, *p* < 0.001) were higher. In the cohort of COPD subjects, 40.0% were “former smokers,” 19.2% were “current smokers”, while 40.8% were “never smokers”, which is consistent with previous analyses of the UK Biobank and attributed to other causes of COPD (i.e., air pollution) [26]. The MLP cohort was more likely to meet criteria for low activity level based on the IPAQ [19] score (18.9% vs 17.4%, *p* < 0.001). The majority of patients identified themselves as current alcohol consumers, although there was a lower proportion in the MLP cohort versus those without MLP (92.4% vs. 93.9%, *p* < 0.001) (Table 1). A sub-analysis of alcohol usage demonstrated a higher proportion of those with MLP who drank alcohol daily/almost daily (26.5% versus 23.8%, *p* < 0.001) (Supp Table 3). Over half (53.4%) of COPD subjects met criteria for MLP using at least one criterion (Supp Table 4, Fig. 1 A,B). A higher proportion of COPD subjects had evidence of MLP based on ASMI and HGS criteria (34% and 26%), followed by FFMI (11%), with the lowest proportion (0.79%) using the BMI criterion.

**Table 1:** Characteristics of adult subjects with COPD from the UK Biobank

	**No MLP criteria** **(*N* = 25,981)**		**MLP criteria** **(*N* = 29,801)**		**Standardized difference (%)**	
**Factor**	**N**	**Statistics**	**N**	**Statistics**		***p*-value**
Sex	25,981		29,801		24.72	< 0.001^c^
Female		13,163 (50.7)		11,464 (38.5)		
Male		12,818 (49.3)		18,337 (61.5)		
Age at recruitment (years)	25,981	57.6 ± 7.8	29,801	60.5 ± 6.9	38.71	< 0.001^a2^
Ethnicity	25,981		29,801		0	< 0.001^c^
British		24,446 (94.1)		28,057 (94.1)		
Irish		653 (2.5)		899 (3.0)		
Any other white background		882 (3.4)		845 (2.8)		
Body mass index (BMI) (Kg/m^2^)	25,981	28.2 ± 4.4	29,743	25.3 ± 4.2	-67.3	< 0.001^a2^
FEV1, percent predicted	25,981	79.7 ± 17.8	29,801	78.7 ± 19.1	-5.5	< 0.001^a2^
FEV1, GOLD stage	25,981		29,801		8.43	< 0.001^c^
> 80% (mild)		13,298 (51.2)		14,842 (49.8)		
50–79% (moderate)		11,242 (43.3)		12,653 (42.5)		
30–49% (severe)		1,266 (4.9)		1,978 (6.6)		
< 30% (very severe)		175 (0.67)		328 (1.1)		
FEV1, liters	25,981	2.4 ± 0.74	29,801	2.4 ± 0.79	-4	< 0.001^a2^
FEV1/FVC ratio	25,981	0.65 ± 0.06	29,801	0.64 ± 0.07	-14.8	< 0.001^a2^
Alcohol drinker status	25,961		29,758		5.45	< 0.001^c^
Never		699 (2.7)		981 (3.3)		< 0.001^d^
Previous		892 (3.4)		1,292 (4.3)		< 0.001^d^
Current		24,370 (93.9)		27,485 (92.4)		< 0.001^d^
Smoking status	25,871		29,656		7.66	< 0.001^c^
Never		10,686 (41.3)		11,981 (40.4)		0.0264^d^
Previous		10,732 (41.5)		11,460 (38.6)		< 0.001^d^
Current		4,453 (17.2)		6,215 (21.0)		< 0.001^d^
Pack years of smoking (years)	11,418	25.5 [14.0, 39.4]	13,407	27.5 [14.8, 42.0]	8.06	< 0.001^b^
Ever addicted to any substance or behavior	7,782	622 (8.0)	8,203	612 (7.5)	-2	0.21^c^
History of cancer diagnosis	25,891	2,001 (7.7)	29,698	2,754 (9.3)	5.54	< 0.001^c^
Doctor diagnosed emphysema	6,010	56 (0.93)	6,425	101 (1.6)	5.76	0.001^c^
Age emphysema diagnosed by doctor	56	54.4 ± 13.1	101	58.7 ± 11.1	35.23	0.032^a1^
IPAQ activity group	21,263		23,966		8.39	< 0.001^c^
Low		3,698 (17.4)		4,539 (18.9)		
Moderate		8,379 (39.4)		9,745 (40.7)		
High		9,186 (43.2)		9,682 (40.4)		
Summed minutes activity (minutes)	21,263	105.0 [60.0, 180.0]	23,966	100.0 [50.0, 180.0]	-6.5	< 0.001^b^
MET minutes per week for walking (minutes/week)	21,263	693.0 [330.0, 1386.0]	23,966	693.0 [330.0, 1386.0]	-1.5	0.12^b^

Statistics presented as Mean ± SD, Median [P25, P75], N (column %)

MLP criteria were defined as meeting at least one of the four MLP definitions

*MET* metabolic equivalent of task, *IPAQ* International Physical Activity Questionnaire, *MLP* muscle loss phenotype

*p*-values:

^a1^t-test

^a2^Satterthwaite t test

^b^Wilcoxon Rank Sum test

^c^Pearson's chi-square test

^d^Fisher's Exact test

### Mortality

A total of 5,608 deaths (10.1%) were observed in the COPD cohort, of which 907 (16.2% of deaths) were related to COPD. Follow-up time ranged between 42 to 82 months with a median of 71 months [P25, P75: 66.6, 75.0]. Average (± SD) age at time of death was 70 ± 6.5 years. Cox regression was performed to determine if COPD subjects with MLP had a higher all-cause mortality rate (Fig. 1 C,D). After adjusting for covariables, the risk of dying was 30% higher for subjects who met any criteria for MLP (HR 1.3, 95% CI: 1.2–1.4). Analyses of the contribution of individual criteria for MLP to all-cause mortality showed that FFMI (HR 1.6, 95%: CI: 1.5–1.8) and BMI (HR 1.6, 95% CI: 1.3–2.0) had the highest association with death, followed by HGS (HR 1.4, 95% CI: 1.3–1.5) and ASMI (HR 1.2, 95% CI: 1.1–1.3) (Table 2). We also found that the hazard of dying due to COPD was 70% higher than other causes of death (HR 1.7, 95% CI: 1.5–2.0) for those with MLP (Fig. 1 E,F). Analyses of individual criteria for MLP showed that BMI (HR 2.2, 95% CI: 1.5–3.3) had the highest association with death, followed by FFMI (HR 2.0, 95% CI: 1.7–2.4), ASMI (HR 1.7, 95% CI: 1.4–2.0), and HGS (HR 1.6, 95% CI: 1.4–1.9) (Table 3, Supp Figure 2).

**Table 2:** MLP criteria and all-cause death: cox regression

Factor	Any MLP criteria		MLP criteria by BMI		MLP criteria by HGS		MLP criteria by FFMI		MLP criteria by ASMI	
	**HR (95% CI)**	***p*-value**	**HR (95% CI)**	***p*-value**	**HR (95% CI)**	***p*-value**	**HR (95% CI)**	***p*-value**	**HR (95% CI)**	***p*-value**
**MLP**	1.3 (1.2, 1.4)	< 0.001	1.6 (1.3, 2.0)	< 0.001	1.4 (1.3, 1.5)	< 0.001	1.6 (1.5, 1.8)	< 0.001	1.2 (1.1, 1.3)	< 0.001
**Male vs. Female**	1.5 (1.4, 1.6)	< 0.001	1.6 (1.6, 1.7)	< 0.001	1.7 (1.6, 1.8)	< 0.001	1.6 (1.6, 1.7)	< 0.001	1.5 (1.4, 1.5)	< 0.001
**Age (years)**	1.09 (1.08,1.09)	< 0.001	1.09 (1.09, 1.10)	< 0.001	1.09 (1.08,1.10)	< 0.001	1.09 s(1.09,1.10)	< 0.001	1.09 (1.09,1.10)	< 0.001
**BMI (1 kg/m**^**2**^** increment)**	1.04 (1.03,1.04)	< 0.001	-	-	1.03 (1.02,1.03)	< 0.001	1.04 (1.03,1.05)	< 0.001	1.04 (1.03,1.05)	< 0.001
**British vs. Other Ethnicity**	0.92 (0.82,1.03)	0.14	0.92 (0.82, 1.02)	0.12	0.93 (0.83,1.03)	0.18	0.92 (0.82,1.03)	0.14	0.91 (0.82,1.02)	0.11
**Cancer diagnosed by doctor**	1.9 (1.7, 2.0)	< 0.001	1.9 (1.7, 2.0)	< 0.001	1.9 (1.7, 2.0)	< 0.001	1.9 (1.7, 2.0)	< 0.001	1.9 (1.7, 2.0)	< 0.001
**Current alcohol use**	0.67 (0.62,0.73)	< 0.001	0.65 (0.60, 0.71)	< 0.001	0.67 (0.62,0.73)	< 0.001	0.67 (0.61,0.73)	< 0.001	0.66 (0.61,0.72)	< 0.001
**Current smoker**	2.4 (2.3, 2.5)	< 0.001	2.3 (2.2, 2.5)	< 0.001	2.4 (2.3, 2.5)	< 0.001	2.4 (2.2, 2.5)	< 0.001	2.4 (2.3, 2.5)	< 0.001
**FEV1/FVC ratio (0.1 unit increment)**	0.73 (0.71,0.76)	< 0.001	0.74 (0.71, 0.76)	< 0.001	0.74 (0.71,0.76)	< 0.001	0.73 (0.71,0.76)	< 0.001	0.73 (0.71,0.75)	< 0.001

All models were fitted on each of the 5 imputed datasets and parameter estimates were combined using SAS Mianalyze. BMI was not included in the model for BMI-defined MLP

*MLP* muscle loss phenotype

**Table 3:** MLP criteria and COPD-related death: competing risk analysis with other-cause death as competing risk

Factor	Any MLP criteria		By BMI		By HGS		By FFMI		By ASMI	
	**HR (95% CI)**	***p*-value**	**HR (95% CI)**	***p*-value**	**HR (95% CI)**	***p*-value**	**HR (95% CI)**	***p*-value**	**HR (95% CI)**	***p*-value**
**MLP**	1.7 (1.5, 2.0)	< 0.001	2.2 (1.5, 3.3)	< 0.001	1.6 (1.4, 1.9)	< 0.001	2.0 (1.7, 2.4)	< 0.001	1.7 (1.4, 2.0)	< 0.001
**Male vs. Female**	1.4 (1.2, 1.7)	< 0.001	1.6 (1.4, 1.9)	< 0.001	1.7 (1.5, 2.0)	< 0.001	1.6 (1.4, 1.9)	< 0.001	1.2 (1.02, 1.4)	0.029
**Age (years)**	1.1 (1.09, 1.1)	< 0.001	1.1 (1.1, 1.1)	< 0.001	1.1 (1.10, 1.1)	< 0.001	1.1 (1.10, 1.1)	< 0.001	1.1 (1.10, 1.1)	< 0.001
**BMI (1 kg/m**^**2**^** increment)**	1.04 (1.03, 1.06)	< 0.001	-	-	1.02 (1.01, 1.04)	0.005	1.05 (1.03, 1.06)	< 0.001	1.06 (1.04, 1.08)	< 0.001
**British vs. Other Ethnicity**	0.85 (0.65, 1.1)	0.240	0.84 (0.65, 1.10)	0.200	0.87 (0.66, 1.1)	0.310	0.86 (0.65, 1.1)	0.250	0.84 (0.64, 1.09)	0.190
**Cancer diagnosed by doctor**	0.98 (0.78, 1.2)	0.88	0.97 (0.77, 1.2)	0.81	0.98 (0.78, 1.2)	0.84	0.99 (0.79, 1.2)	0.96	0.99 (0.79, 1.2)	0.92
**Current alcohol use**	0.62 (0.50, 0.76)	< 0.001	0.60 (0.49, 0.74)	< 0.001	0.62 (0.51, 0.77)	< 0.001	0.60 (0.49, 0.73)	< 0.001	0.61 (0.49, 0.74)	< 0.001
**Current smoker**	4.6 (4.0, 5.2)	< 0.001	4.4 (3.8, 5.0)	< 0.001	4.6 (4.0, 5.2)	< 0.001	4.5 (3.9, 5.1)	< 0.001	4.5 (4.0, 5.2)	< 0.001
**FEV1/FVC ratio (0.1 unit increment)**	0.45 (0.42, 0.47)	< 0.001	0.45 (0.43, 0.47)	< 0.001	0.45 (0.42, 0.47)	< 0.001	0.44 (0.42, 0.47)	< 0.001	0.44 (0.42, 0.47)	< 0.001

All models were fitted on each of the 5 imputed datasets and parameter estimates were combined using SAS Mianalyze. BMI was not included in the model for BMI-defined MLP criteria

*MLP* muscle loss phenotype

### Hospitalizations

**Fig. 2:**
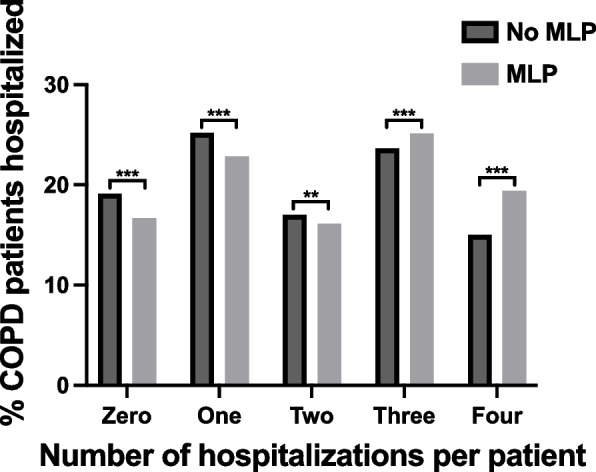
A higher number of hospitalizations was associated with muscle loss phenotype in patients with COPD. The proportion of COPD patients who were hospitalized (from zero to four times) between their initial UK biobank assessment and follow-up period. The y-axis represents the percent of COPD patients who were hospitalized. The bar graphs represent no muscle loss phenotype (MLP) versus those with MLP. The x-axis represents the number of hospitalizations (*n* = 0-4). COPD patients without MLP were more likely to be hospitalized 0-2 times while those with MLP were more likely to be hospitalized 3-4 times

Subjects with MLP were more likely to be hospitalized (19.7% higher odds [adjusted OR = 1.197, CI: 1.184–1.210]). MLP was also less common in those who avoided hospitalization (adjusted OR 0.88, CI: 0.82–0.94) (Supp Table 5). When compared to COPD patients without MLP, a greater proportion of COPD patients with MLP reported three (25.1% versus 23.6%, *p* < 0.001) or four (19.4% versus 15.0%, *p* < 0.001) hospitalizations during the follow-up period (Fig. 2, Supp Table 6).

## Discussion

In a large population-based cohort of subjects from the UK Biobank with COPD, over half (53.4%) had evidence of MLP, with the greatest proportion having low HGS (26.3%) or low ASMI (33.8%). We further noted increased risk of all-cause or COPD-related death associated with MLP, after adjustment for covariables including severity of COPD.

Interestingly, we noted that the prevalence of MLP based on BMI and FFMI criteria represented a lower proportion of subjects, whereas other criteria including HGS and ASMI represented a higher proportion of subjects. BMI and FFMI were more predictive of all-cause or COPD-related death as compared to HGS and ASMI. These findings suggest that BMI and FFMI may predict a more severe cohort of MLP. However, we note that the BMI criterion was the lowest proportion of COPD patients in our analysis (0.79%). Given that other measures of MLP were more prevalent and predicted adverse outcomes, low BMI may represent a specific but not sensitive way to diagnose MLP in COPD patients.

Others have reported that loss of muscle strength occurs more rapidly than muscle mass, suggesting that loss of muscle strength predicts a decline in muscle quality [15]. Measuring HGS is increasingly being recognized as a simple bedside procedure that can be incorporated into clinical practice to consider the diagnosis of muscle dysfunction [27, 28]. Our findings highlight that reductions in HGS are common in a significant proportion of COPD subjects and remains strongly associated with adverse clinical outcomes. When considering when to screen clinically, many clinicians use the SARC-F questionnaire to screen for symptoms of sarcopenia, which can then be followed by additional testing for muscle strength, muscle mass, or physical performance (i.e. HGS, FFMI/ASMI, sit-to-stand test, six minute walk test) in at-risk patients [29]. This may be a practical and efficient approach for clinicians to identify at-risk COPD patients who may benefit from additional testing in their clinical practice.

The UK Biobank has previously been considered to have a “healthy volunteer” effect in the subjects recruited [30]. This was consistent with our findings which demonstrated that the average COPD patient was early stage COPD (either “mild” or “moderate” severity of obstruction per the GOLD guidelines [31]). However, despite this, we noted a high proportion of COPD patients demonstrated evidence for MLP at the population-level which was independently associated with risk for all-cause and COPD-related death. While similar population-based studies (i.e. Rotterdam, MESA) have noted an association of COPD and MLP [32, 33], they did not incorporate or compare multiple anthropometric measures in the same sample [33], or analyze risk for adverse outcomes [32, 33]. The Copenhagen Heart Study identified that both low FFMI and low BMI were associated with mortality in COPD patients, but did not incorporate concurrent comparisons of low ASMI or low HGS in their analyses [12]. We believe our more comprehensive analyses, which compared multiple anthropometric measures in the same sample, allows for the disease trajectory of MLP to be captured, and identifies how certain criteria are more predictive of adverse outcomes than others.

While our analysis identified novel findings in a large, population based cohort, we recognize several limitations of our study. Our definition of COPD included a pre-bronchodilator FEV1/FVC ratio < 0.7, given that post-bronchodilator spirometry is not available in the UK Biobank. While there is potential to over-diagnose COPD when using pre-bronchodilator testing [34], pre-bronchodilator testing has been previously used in UK Biobank studies of COPD [35, 36] and other large scale epidemiologic studies including the Copenhagen Heart Study [12]. Even though the UK Biobank provides the age at which COPD was diagnosed by a physician, the true duration of COPD diagnosis may be longer. In human studies, it is always challenging to identify the “true” onset of disease because of lead time bias inherent in diagnoses. In this population known for a selection bias of “healthy volunteers”, we also noted a significant proportion (40.8%) of COPD patients reported being “never smokers.” While tobacco smoke is the most common risk factor for COPD, previous population-level studies of the UK Biobank have confirmed that a significant number of COPD patients are “never-smokers” which has been attributed to other causes such as air pollution [26]. Recent guidelines have also highlighted the prevalence and impact of non-smoking causes for COPD [17]. Interestingly, we found an association of alcohol use in our COPD cohort with daily or almost daily ethanol consumption was associated with MLP. Alcohol consumption is a well-known cause for loss of muscle mass [37], and our data highlight that multiple factors may contribute to MLP in patients with COPD. Aging-related loss of muscle mass and function is common in patients after age 50 years [38] and the average age of COPD patients in our study was ~ 60 years. However, others have reported that muscle loss is accelerated in COPD patients when compared to a healthy aged population [39]. Also, muscle loss or dysfunction occurs in up to 40% of COPD patients [7, 12, 40–42], which is consistent with the proportion of patients with MLP in our analysis. In contrast, aging-related muscle loss represents approximately 10% of patients older than 60 years [23, 43, 44]. These data show that despite the age of the patients in our cohort, MLP is higher than expected in a population of a comparable age without COPD. Finally, while MLP was associated with adverse all-cause and COPD related outcomes in the UK Biobank, there remain a number of gaps in our understanding of the underlying mechanisms that cause muscle loss and dysfunction in COPD [39]. Our preclinical studies in skeletal muscle cells using an in vitro model of prolonged intermittent hypoxia demonstrate physiologic perturbations consistent with muscle loss in COPD [45]. We have also identified single nucleotide polymorphisms in the UK Biobank associated with low muscle mass in COPD patients, allowing for further translation of these preclinical data to human studies [35]. Additional studies are needed to determine the molecular mechanisms of muscle loss and dysfunction in COPD to develop new and innovative therapeutic strategies for COPD patients with MLP.

## Conclusion

Our analysis of the UK Biobank identified that evidence of MLP is common in a large population-based cohort of COPD patients based on multiple criteria (FFMI, ASMI, HGS, BMI) and is associated with adverse clinical outcomes. Our data lay the foundation for future studies and targeted interventions for MLP in COPD and the need to develop clinically applicable criteria for MLP in chronic diseases beyond COPD.

### Supplementary Information

**Supplementary Material 1.**

**Supplementary Material 2.**

## Data Availability

The data that support the findings of this study are available from UK Biobank ( https://www.ukbiobank.ac.uk/), which is subject to successful registration and application process.

## Abbreviations

ASMI: Appendicular skeletal muscle index
BMI: Body mass index
COPD: Chronic obstructive pulmonary disease
FEV1: Forced expiratory volume1
FFMI: Fat-free mass index
FVC: Forced vital capacity
GOLD: Global Initiative for Chronic Obstructive Lung Disease
HGS: Handgrip strength
HR: Hazard ratio
IPAQ: International Physical Activity Questionnaire
OR: Odds ratio
UK: United Kingdom
