# Supplementary material for: Muscle loss phenotype in COPD is associated with adverse outcomes in the UK Biobank
Source: BMC Pulm Med. 2024 Apr 17;24:186. doi: 10.1186/s12890-024-02999-7 (PMC11025247; doi:10.1186/s12890-024-02999-7)
Supplement: Supplementary file 1 — Supplementary Material 1. [file 12890_2024_2999_MOESM1_ESM.docx]

**Supplementary Tables**

**Supplementary Table 1. Definitions for muscle loss phenotype (MLP).**

**Supplementary Table 2. ICD-CM codes used to classify hospitalization diagnoses.**

**Supplementary Table 3. Sub-analysis of alcohol usage in Adult Subjects with COPD from the UK Biobank.**

**Supplementary Table 4. Summary of Tests Used to Define Evidence for Muscle Loss Phenotype (MLP).**

**Supplementary Table 5. Evidence of muscle loss phenotype (MLP) and Number of Hospitalizations: Zero-Inflated Negative Binomial Regression.**

**Supplementary Table 6. Cause for hospitalization in those with evidence of versus those without evidence of muscle loss phenotype (MLP).**

**Supplementary Table 1. Definitions for muscle loss phenotype**

| **Muscle loss phenotype was defined either as:** |
| --- |
| 1. Low muscle mass (whole body). FFMI (fat free mass index): Whole body fat free mass / height (m)^2^ <17.4 kg/m2 for male subjects and <15 kg/m^2^ for female subjects as determined by bioelectric impedance^1^. 2. Low muscle mass (appendages). ASMI (appendicular skeletal muscle index): Sum of the muscle mass (fat free mass) of the 4 limbs / height (m)^2^. ASMI < 8.90 kg/m^2^ in men and < 6.37 kg/m^2^ in women^2^. 3. Low muscle strength. HGS (handgrip strength): <30 kg for men and <20 kg for women^3^. 4. Low muscle and fat mass. BMI (body mass index): <18.5 kg/m^2 4^. While low BMI has traditionally been considered as evidence for malnutrition^5^, it is the most common method that physicians consider muscle loss clinically and has been incorporated into COPD prognostication^6^, and therefore was included in our analysis. |

**Supplementary Table 2. ICD-CM codes used to classify hospitalization diagnoses**

| **Disease** | **ICD-9-CM** | **ICD-10-CM** |
| --- | --- | --- |
| COPD | 491.**, 492.0. 492.8, 493.20, 493.21, 493.22, 496.* | J42; J41.0; J41.1, J41.8; J44.9; J44.1; J44.0; J41.8; J42; J43.0, J43.1, J43.2; J43.9; J43.8 |
| Malnutrition | 260, 261, 262, 263.0, 263.1, 263.8, 263.9, 783.21, 783.41, 783.7, 783.9 | E40-E43, E44.0, E44.1, E46, R62.51, R62.7, R63.4, R63.8 |
| Primary pulmonary hypertension | 416 | I27.0 |
| Secondary pulmonary hypertension | 416.1, 416.8 | I27.1, I27.2, I27.89, |
|  |  | I27.20- I27.24, I27.29, I27.83s |
| Pulmonary hypertension due to lung diseases | 416.8, 416.9 | 127.2, I27.23, I27.81 |
| Chronic thromboembolic pulmonary hypertension | 416.8 | 127.2, I27.24 |
| Pulmonary hypertension due to heart disease | 416.8, 416.9, 745.4 | I27.9, I27.22, I27.83, I27.2, I27.89 |
| Acute respiratory distress syndrome | 518.82 | J80 |
| Pulmonary embolism | 415.0, 415.12, 415.13, 415.19 | I26.99, I26.94, I26.93, I26.92 ; I26.90; I26.09 ; I26.02 ; I26.01 |
| Sepsis | 995.91, 995.92 - 995.94, | A41**, R65** |
| Pneumonia | 480.0-480.3, 480.8, 480.9, 481, 482.0-482.2,482.31-482.32, 482.39, 482.40-482.42, 482.49, 482.81-482.83, 482.89, 482.9, 483.0-483.1, 483.8, 484.7, 484.8, 485, 486, 487.0, 514, 517.1 | J12**, J13, J14, J15**, J16**, J17, J18** |
| Shock state | 040.82, 785.50, 785.51, 785.59, 785.52 + 995.92 | R57**, R65.21, T79.4, T78.2, T78.0, T80.5, T75.4, A48.3, T81.1, 3E030XZ, 3E040XZ |
| Acute kidney failure | 584.5-584.9 | N17** |
| Acute liver failure | 573.3, 570, | K720*, K712 |
|  | 570 + 572.2 |  |

**Supplementary Table 3. Sub-analysis of alcohol usage in Adult Subjects with COPD from the UK Biobank**

|  | **No MLP (N=25,981)** | | **MLP (N=29,801)** | | **Standardized Difference (%)** |  |
| --- | --- | --- | --- | --- | --- | --- |
| **Factor** | **N** | **Statistics** | **N** | **Statistics** |  | **p-value** |
| Alcohol intake frequency | 25,963 |  | 29,765 |  | 8.39 | <0.001^c^ |
| Daily or almost daily |  | 6,182 (23.8) |  | 7,878 (26.5) |  | <0.001^d^ |
| Three or four times a week |  | 6,289 (24.2) |  | 6,779 (22.8) |  | <0.001^d^ |
| Once or twice a week |  | 6,549 (25.2) |  | 6,948 (23.3) |  | <0.001^d^ |
| One to three times a month |  | 2,676 (10.3) |  | 2,687 (9.0) |  | <0.001^d^ |
| Special occasions only |  | 2,674 (10.3) |  | 3,193 (10.7) |  | 0.105 |
| Never |  | 1,593 (6.1) |  | 2,280 (7.7) |  | <0.001^d^ |
| Alcohol drinker status | 25,961 |  | 29,758 |  | 5.45 | <0.001^c^ |
| Never |  | 699 (2.7) |  | 981 (3.3) |  | <0.001^d^ |
| Previous |  | 892 (3.4) |  | 1,292 (4.3) |  | <0.001^d^ |
| Current |  | 24,370 (93.9) |  | 27,485 (92.4) |  | <0.001^d^ |
| Amount of alcohol drunk on a typical drinking day | 7,244 |  | 7,545 |  | 13.69 | <0.001^c^ |
| 1 or 2 |  | 3,467 (47.9) |  | 3,903 (51.7) |  | 0.390 |
| 3 or 4 |  | 1,986 (27.4) |  | 2,007 (26.6) |  | <0.001^d^ |
| 5 or 6 |  | 950 (13.1) |  | 924 (12.2) |  | <0.001^d^ |
| 7, 8 or 9 |  | 563 (7.8) |  | 496 (6.6) |  | <0.001^d^ |
| 10 or more |  | 278 (3.8) |  | 215 (2.8) |  | <0.001^d^ |

Statistics presented as Median [P25, P75], N (column %).p-values: b=Wilcoxon Rank Sum test, c=Pearson's chi-square test, d=Fisher's Exact test.

MLP (muscle loss phenotype) is defined as meeting at least one of the 4 MLP definitions

**Supplementary Table 4. Summary of Tests Used to Define Evidence for MLP**

|  | **No evidence of muscle wasting** | | **Evidence of muscle wasting** | |
| --- | --- | --- | --- | --- |
| **Definition for MLP** | **N** | **Statistics** | **N** | **Statistics** |
| BMI (kg/m^2^) | 55,281 | 99.21% | 443 | 0.79% |
| Fat free mass index [FFMI] (kg/m^2^) | 49,636 | 89.0% | 6,146 | 11.0% |
| Handgrip strength [HGS] (kg) | 41,027 | 73.7% | 14,646 | 26.3% |
| Appendicular skeletal muscle index [ASMI] (kg/m^2^) | 36,948 | 66.2% | 18,834 | 33.8% |
| Any criteria | 25,981 | 46.6% | 29,801 | 53.4% |

MLP criteria defined by BMI as <18.5 kg/m^2^, FFMI <17.4 kg/m2 for male and <15 kg/m^2^ for female, ASMI <8.9 kg/m^2^ for males and <6.37 kg/m^2^ for females. HGS<30 kg for males and <20 kg for females.

**Supplementary Table 5. Evidence of MLP and Number of Hospitalizations: Zero-Inflated Negative Binomial Regression**

| **Factor** | **Any MLP criteria** | | **By BMI** | | **By HGS** | | **By FFMI** | | **By ASMI** | |
| --- | --- | --- | --- | --- | --- | --- | --- | --- | --- | --- |
|  |  | **p-value** |  | **p-value** |  | **p-value** |  | **p-value** |  | **p-value** |
| **Negative Binomial Model Explaining Num. of Hospitalizations** | | | | | | | | | | |
| MLP criteria | 1.197 (1.184, 1.210) | <0.001 | 1.206 (1.149, 1.265) | <0.001 | 1.263 (1.244, 1.284) | <0.001 | 1.308 (1.287, 1.329) | <0.001 | 1.119 (1.104, 1.135) | <0.001 |
| Male vs. Female | 1.019 (1.009, 1.030) | <0.001 | 1.059 (1.048, 1.070) | <0.001 | 1.091 (1.080, 1.103) | <0.001 | 1.059 (1.049, 1.071) | <0.001 | 0.993 (0.982, 1.005) | 0.270 |
| Age (years) | 1.008 (1.007, 1.00) | <0.001 | 1.010 (1.010, 1.011) | <0.001 | 1.008 (1.007, 1.008) | <0.001 | 1.009 (1.009, 1.010) | <0.001 | 1.009 (1.008, 1.010) | <0.001 |
| BMI (1 Kg/m2 increment) | 1.022 (1.021, 1.023) | <0.001 | --- | --- | 1.016 (1.015, 1.017) | <0.001 | 1.023 (1.022, 1.024) | <0.001 | 1.022 (1.021, 1.023) | <0.001 |
| British vs. Other Ethnicity | 0.941 (0.921, 0.961) | <0.001 | 0.939 (0.919, 0.959) | <0.001 | 0.947 (0.927, 0.967) | <0.001 | 0.938 (0.918, 0.958) | <0.001 | 0.936 (0.917, 0.956) | <0.001 |
| Cancer diagnosed by doctor | 2.271 (2.244, 2.300) | <0.001 | 2.266 (2.239, 2.295) | <0.001 | 2.271 (2.243, 2.299) | <0.001 | 2.275 (2.248, 2.303) | <0.001 | 2.277 (2.249, 2.305) | <0.001 |
| Current alcohol use | 0.786 (0.773, 0.800) | <0.001 | 0.767 (0.754, 0.780) | <0.001 | 0.791 (0.778, 0.805) | <0.001 | 0.780 (0.767, 0.793) | <0.001 | 0.778 (0.765, 0.791) | <0.001 |
| Current smoker | 1.184 (1.167, 1.201) | <0.001 | 1.168 (1.152, 1.185) | <0.001 | 1.184 (1.167, 1.201) | <0.001 | 1.180 (1.164, 1.197) | <0.001 | 1.186 (1.169, 1.203) | <0.001 |
| FEV1/FVC ratio (0.1 unit increment) | 0.932 (0.925, 0.939) | <0.001 | 0.930 (0.924, 0.937) | <0.001 | 0.935 (0.928, 0.942) | <0.001 | 0.931 (0.925, 0.938) | <0.001 | 0.928 (0.921, 0.935) | <0.001 |
| **Logit Model Explaining Avoidance of Hospitalization** | | | | | | | | | | |
| MLP criteria | 0.877 (0.823, 0.936) | <0.001 | 1.253 (0.935, 1.678) | 0.130 | 0.826 (0.771, 0.886) | <0.001 | 0.881 (0.802, 0.968) | 0.009 | 0.895 (0.828, 0.967) | 0.005 |
| Male vs. Female | 1.306 (1.230, 1.387) | <0.001 | 1.171 (1.106, 1.239) | <0.001 | 1.227 (1.156, 1.303) | <0.001 | 1.244 (1.173, 1.320) | <0.001 | 1.324 (1.237, 1.418) | <0.001 |
| Age (years) | 0.956 (0.952, 0.959) | <0.001 | 0.953 (0.949, 0.956) | <0.001 | 0.955 (0.952, 0.959) | <0.001 | 0.954 (0.951, 0.958) | <0.001 | 0.955 (0.952, 0.958) | <0.001 |
| BMI (1 Kg/m2 increment) | 0.941 (0.934, 0.948) | <0.001 | --- | --- | 0.949 (0.942, 0.955) | <0.001 | 0.945 (0.938, 0.952) | <0.001 | 0.942 (0.934, 0.950) | <0.001 |
| British vs. Other Ethnicity | 0.773 (0.694, 0.861) | <0.001 | 0.779 (0.701, 0.867) | <0.001 | 0.770 (0.691, 0.858) | <0.001 | 0.776 (0.696, 0.864) | <0.001 | 0.777 (0.698, 0.865) | <0.001 |
| Cancer diagnosed by doctor | 0.320 (0.278, 0.367) | <0.001 | 0.320 (0.279, 0.368) | <0.001 | 0.322 (0.280, 0.370) | <0.001 | 0.319 (0.277, 0.367) | <0.001 | 0.317 (0.276, 0.365) | <0.001 |
| Current alcohol use | 1.341 (1.189, 1.512) | <0.001 | 1.384 (1.231, 1.557) | <0.001 | 1.332 (1.181, 1.502) | <0.001 | 1.346 (1.195, 1.517) | <0.001 | 1.350 (1.198, 1.521) | <0.001 |
| Current smoker | 0.682 (0.633, 0.736) | <0.001 | 0.709 (0.658, 0.763) | <0.001 | 0.684 (0.634, 0.737) | <0.001 | 0.680 (0.631, 0.733) | <0.001 | 0.682 (0.633, 0.736) | <0.001 |
| FEV1/FVC ratio (0.1 unit increment) | 1.057 (1.011, 1.105) | 0.016 | 1.060 (1.014, 1.107) | 0.010 | 1.054 (1.008, 1.103) | 0.022 | 1.058 (1.011, 1.106) | 0.014 | 1.059 (1.013, 1.107) | 0.012 |

Age represents the age when attending the assessment center.

**Supplementary Table 6. Cause for hospitalization in those with evidence of MLP versus those without evidence of MLP**

|  | **No evidence of MLP (N=25,981)** | | **Evidence of MLP (N=29,801)** | |  |  |
| --- | --- | --- | --- | --- | --- | --- |
| **Factor** | **N** | **Statistics** | **N** | **Statistics** | **Standardized Difference (%)** | **p-value** |
| Visits to the hospital | 21,501 | 2.0 [1.00, 4.0] | 24,934 | 2.0 [1.00, 4.0] | 10.91 | <0.001^b^ |
| Visits to the hospital (by number) | 21,501 |  | 24,934 |  | 11.99 | <0.001^b^ |
| Zero |  | 4,116 (19.1) |  | 4,155 (16.7) | 2.40 | <0.001^e^ |
| One |  | 5,418 (25.2) |  | 5,681 (22.8) | 2.40 | <0.001^e^ |
| Two |  | 3,652 (17.0) |  | 4,021 (16.1) | 0.90 | 0.004^e^ |
| Three |  | 5,084 (23.6) |  | 6,248 (25.1) | 1.50 | <0.001^e^ |
| Four |  | 3,231 (15.0) |  | 4,829 (19.4) | 4.40 | <0.001^e^ |
| COPD-related | 21,858 | 1,979 (9.1) | 25,635 | 3,411 (13.3) | 13.52 | <0.001^c^ |
| Malnutrition-related | 21,858 | 454 (2.1) | 25,635 | 1,004 (3.9) | 10.8 | <0.001^c^ |
| Pulmonary HTN-related | | | | | | |
| Primary pulmonary HTN | 21,858 | 35 (0.16) | 25,635 | 65 (0.25) | 2.06 | 0.027^c^ |
| Secondary pulmonary HTN | 21,858 | 58 (0.27) | 25,635 | 97 (0.38) | 2 | 0.031^c^ |
| Pulmonary HTN due to  lung diseases | 21,858 | 57 (0.26) | 25,635 | 96 (0.37) | 2.02 | 0.029^c^ |
| Chronic thromboembolic  pulmonary HTN | 21,858 | 57 (0.26) | 25,635 | 96 (0.37) | 2.02 | 0.029^c^ |
| Pulmonary HTN due to  heart disease | 21,858 | 73 (0.33) | 25,635 | 125 (0.49) | 2.4 | 0.010^c^ |
| In-hospital diagnoses | | | | | | |
| Acute respiratory distress syndrome | 21,858 | 13 (0.06) | 25,635 | 25 (0.10) | 1.36 | 0.14^c^ |
| Pulmonary embolism | 21,858 | 358 (1.6) | 25,635 | 431 (1.7) | 0.34 | 0.71^c^ |
| Sepsis | 21,858 | 412 (1.9) | 25,635 | 589 (2.3) | 2.88 | 0.002^c^ |
| Pneumonia | 21,858 | 1,033 (4.7) | 25,635 | 1,894 (7.4) | 11.18 | <0.001^c^ |
| Shock state | 21,858 | 77 (0.35) | 25,635 | 103 (0.40) | 0.81 | 0.38^c^ |
| Acute kidney failure | 21,858 | 609 (2.8) | 25,635 | 943 (3.7) | 5.05 | <0.001^c^ |
| Acute liver failure | 21,858 | 9 (0.04) | 25,635 | 15 (0.06) | 0.78 | 0.40^c^ |

Statistics presented as Median [P25, P75], N (column %). p values: b=Wilcoxon Rank Sum test, c=Pearson's chi-square test, d=Fisher's Exact test, e= Z score test of proportions. HTN = hypertension.

MLP (muscle loss phenotype) is defined as meeting at least one of the 4 sarcopenia definitions.

**References:**

1 Gonzalez MC, Pastore CA, Orlandi SP, Heymsfield SB. Obesity paradox in cancer: new insights provided by body composition. Am J Clin Nutr. 2014; **99**: 999-1005.

2 da Silva Alexandre T, de Oliveira Duarte YA, Ferreira Santos JL, Wong R, Lebrao ML. Sarcopenia according to the european working group on sarcopenia in older people (EWGSOP) versus Dynapenia as a risk factor for disability in the elderly. J Nutr Health Aging. 2014; **18**: 547-53.

3 Lauretani F, Russo CR, Bandinelli S, Bartali B, Cavazzini C, Di Iorio A, Corsi AM, Rantanen T, Guralnik JM, Ferrucci L. Age-associated changes in skeletal muscles and their effect on mobility: an operational diagnosis of sarcopenia. J Appl Physiol (1985). 2003; **95**: 1851-60.

4 Evans WJ, Morley JE, Argilés J, Bales C, Baracos V, Guttridge D, Jatoi A, Kalantar-Zadeh K, Lochs H, Mantovani G, Marks D, Mitch WE, Muscaritoli M, Najand A, Ponikowski P, Rossi Fanelli F, Schambelan M, Schols A, Schuster M, Thomas D, Wolfe R, Anker SD. Cachexia: a new definition. Clin Nutr. 2008; **27**: 793-9.

5 Cederholm T, Bosaeus I, Barazzoni R, Bauer J, Van Gossum A, Klek S, Muscaritoli M, Nyulasi I, Ockenga J, Schneider SM, de van der Schueren MA, Singer P. Diagnostic criteria for malnutrition - An ESPEN Consensus Statement. Clin Nutr. 2015; **34**: 335-40.

6 Celli BR, Cote CG, Marin JM, Casanova C, Montes de Oca M, Mendez RA, Pinto Plata V, Cabral HJ. The body-mass index, airflow obstruction, dyspnea, and exercise capacity index in chronic obstructive pulmonary disease. N Engl J Med. 2004; **350**: 1005-12.
