# Supplementary material for: Muscle loss phenotype in COPD is associated with adverse outcomes in the UK Biobank
Source: BMC Pulm Med. 2024 Apr 17;24:186. doi: 10.1186/s12890-024-02999-7 (PMC11025247; doi:10.1186/s12890-024-02999-7)
Supplement: Supplementary file 2 — Supplementary Material 2. [file 12890_2024_2999_MOESM2_ESM.zip › Supplementary Fig Legends 3.19.24 AA no marked up.docx]

**Supplementary Figure Legends**

**Supplementary Figure 1. Consort diagram of subjects with COPD selected from the UK biobank database.**

**Supplementary Figure 2. Cox-regression analysis of individual criteria for MLP and all-cause death.** Each criteria for MLP was independently associated (p<0.001) with all-cause death, including appendicular skeletal muscle index(ASMI), fat free mass index(FFMI), body mass index(BMI), and handgrip strength (HGS).
