# Supplementary figures and images for: Muscle loss phenotype in COPD is associated with adverse outcomes in the UK Biobank
Source: BMC Pulm Med. 2024 Apr 17;24:186. doi: 10.1186/s12890-024-02999-7 (PMC11025247; doi:10.1186/s12890-024-02999-7)

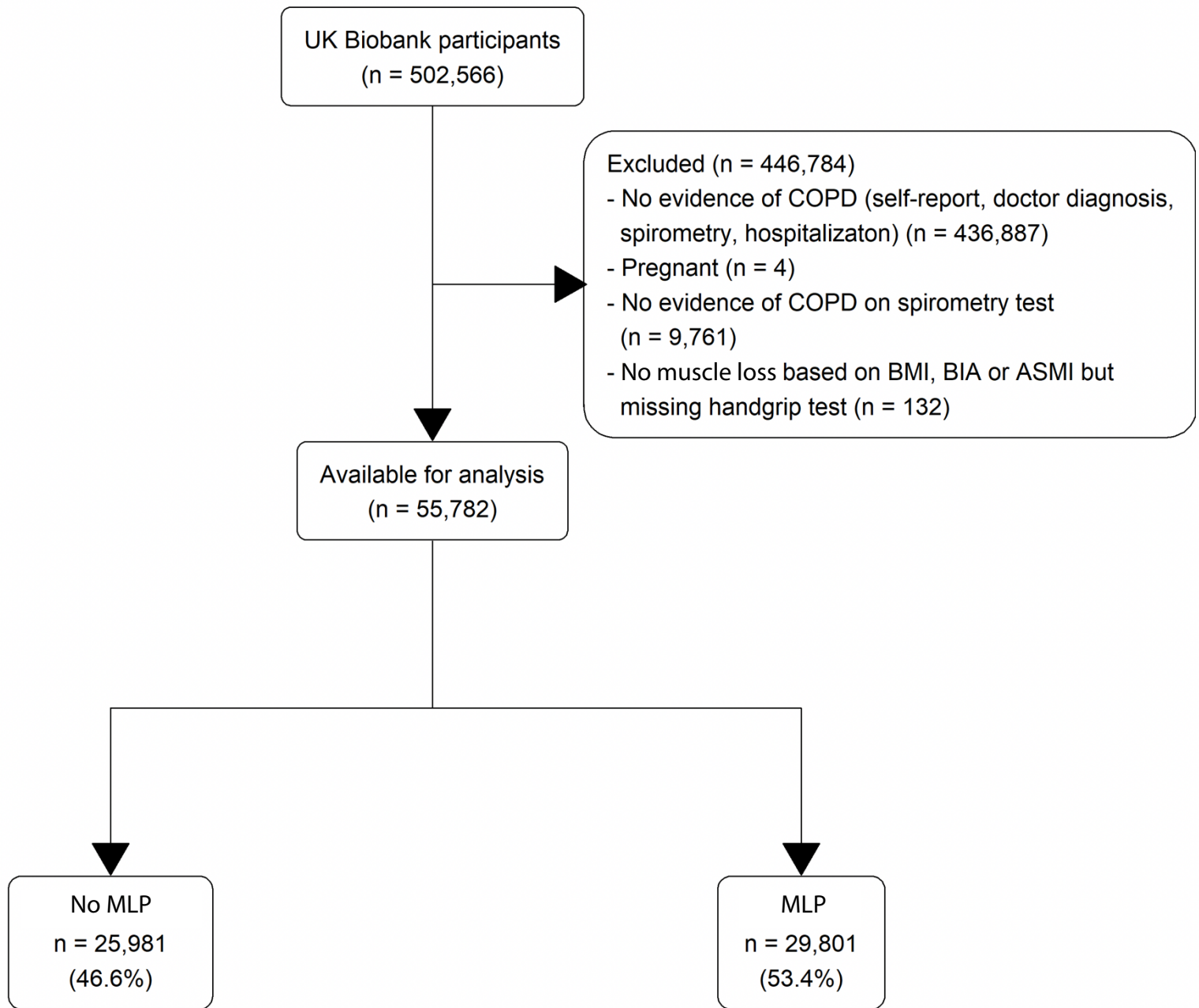

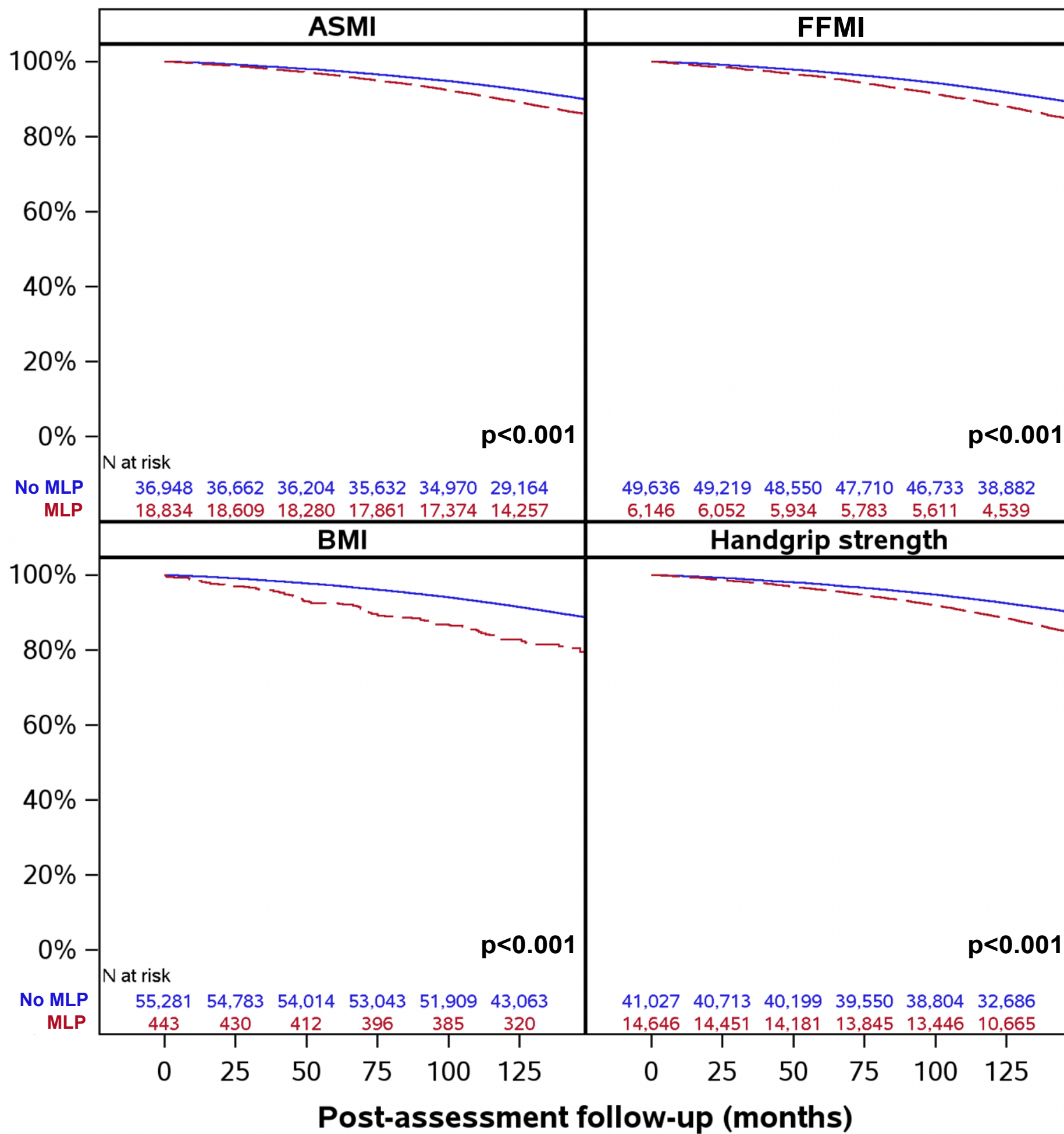

Supplement: Supplementary file 2 — Supplementary Material 2. [file 12890_2024_2999_MOESM2_ESM.zip › supp fig 1, 2 3.11.24.pdf]
